# A Comparative Study of Neutron Shielding Performance in Al-Based Composites Reinforced with Various Boron-Containing Particles for Radiotherapy: A Monte Carlo Simulation

**DOI:** 10.3390/nano14211696

**Published:** 2024-10-23

**Authors:** Shiyan Yang, Yupeng Yao, Hanlong Wang, Hai Huang

**Affiliations:** 1 Key Laboratory of Material Physics, Ministry of Education, School of Physics, Zhengzhou University, Zhengzhou 450001, China; shiyanyang@alu.fudan.edu.cn (S.Y.);; 2 Institute of Modern Physics, Fudan University, Shanghai 200433, China; 3 Department of Radiation Oncology, Qilu Hospital of Shangdong University, Jinan 250012, China

**Keywords:** boron-containing Al-based composites, neutron shielding, ambient dose equivalent, Monte Carlo simulation

## Abstract

This study aimed to assess and compare the shielding performance of boron-containing materials for neutrons generated in proton therapy and used in boron neutron capture therapy (BNCT). Five composites, including AlB_2_, Al-B_4_C, Al-TiB_2_, Al-BN, and Al-TiB_2_-BN, were selected as shielding materials, with concrete used as a benchmark. The mass fraction of boron compounds in these materials ranged from 10% to 50%. The Monte Carlo toolkit Geant4 was employed to calculate shielding parameters, including neutron ambient dose equivalent, dose values, and macroscopic cross-section. Results indicated that, compared to concrete, these boron-containing materials more effectively absorb thermal neutrons. When the boron compound exceeds 30 wt.%, these materials exhibit better shielding performance than concrete of the same thickness for neutrons generated by protons. For a given material, its shielding capability increases with boron content. Among the five materials when the material thickness and boron compound content are the same, the shielding performance for neutrons generated by protons, from best to worst, is as follows: Al-TiB_2_, Al-B_4_C, AlB_2_, Al-TiB_2_-BN, and Al-BN. For BNCT, the shielding performance from best to worst is in the following order: Al-B_4_C, AlB_2_, Al-TiB_2_, Al-TiB_2_-BN, and Al-BN. The results of this study provide references and guidelines for the selection and optimization of neutron shielding materials in proton therapy and BNCT facilities.

## 1. Introduction

Recent advancements in radiotherapy technology have led to the increasing prominence and widespread adoption of emerging particle therapies, such as proton and heavy ion radiotherapy [1,2], as well as boron neutron capture therapy (BNCT) [3]. Compared to conventional X-ray radiotherapy, these techniques offer several advantages, including enhanced biological effects and improved protection of surrounding healthy tissues.

Despite these advanced treatment techniques providing many advantages, all of them face a common challenge: neutron radiation shielding [4,5]. In proton and heavy ion radiotherapy, neutrons are primarily produced through the inelastic interactions between primary particles and atomic nuclei. These neutrons, which have high energy and a broad energy distribution (e.g., for the highest energy of 250 MeV used in therapeutic protons, the energy of neutrons generated by the protons at the angle 0° relative to the beam axis ranges from a few eV to 200 MeV [4]), contribute a lower percentage to the therapeutic target dose compared to primary particles. However, their presence increases radiation dose levels for the public and occupational workers, posing a significant risk. In BNCT, neutrons are used directly as the primary particle source for tumor irradiation. Traditionally, reactors were employed to provide neutron sources with energies around 10 MeV. Recent developments have applied accelerators for neutron generation, which have lowered both the maximum and average neutron energies, thus offering several benefits to BNCT treatment. Nevertheless, neutrons still have a maximum energy of a few MeV, continuing to present challenges for effective shielding.

Neutrons pose a significant health hazard due to their higher relative biological effectiveness and have a greater range in matter compared to charged particles of the same energy. Consequently, more effective materials are needed for adequate shielding. Currently, concrete walls are commonly used for shielding in radiotherapy settings, such as hospitals, due to their ease of processing and cost-effectiveness. However, they present several issues, including susceptibility to cracking, insufficient shielding efficiency, and the need for greater thickness to achieve adequate protection [6,7]. To meet shielding requirements, recent research has focused on boron-containing aluminum (Al)-based materials [8,9,10], which leverage aluminum’s unique properties—such as lightweight, high strength, and high wear resistance—combined with boron’s excellent neutron shielding performance. Common examples include Al-B_4_C [8,9], AlB_2_ [11], and Al-TiB_2_ [12,13]. Titanium (Ti) is often added to enhance interfacial properties and increase the strength of aluminum [13]. These materials have been widely adopted in neutron radiation shielding, and their shielding performance for neutrons has been investigated and compared. For instance, Qi et.al. [10] reviewed the advantages and disadvantages of neutron shielding metal alloy materials, such as B_4_C/Al and B/Al alloy, for the storage of spent nuclear fuel. Yusupov et.al [14] manufactured a highly borated (~50 wt.%) dispersed boron–aluminum composite and assessed its neutron-shielding properties. Jia et al. [15] investigated the neutron shielding and mechanical properties of a short carbon fiber-reinforced B_4_C/Al hybrid composite. Lee et.al [16] evaluated the mechanical and thermal neutron absorbing properties of B4C/Al alloy composites.

The purpose of this study is to evaluate and compare the neutron shielding performance of boron-containing Al-based materials in radiotherapy settings, including proton therapy and BNCT, and to explore their potential applications in enhancing neutron shielding in radiotherapy. Due to the limitations of performing measurements only in specialized facilities with well-prepared shielding materials, this study employs Monte Carlo (MC) simulations to assess the shielding effectiveness.

## 2. Materials and Methods

All simulations presented in this work were conducted using the Geant4 (version 10.1.2) MC toolkit [17,18]. The G4EmStandardPhysics_option4 and QGSP_BIC_HP physics lists were used to handle electromagnetic and hadronic interactions, respectively. In the hadronic physic list, the neutron high precision (HP) package was applied to handle neutron interactions from thermal energies up to 20 MeV.

To assess and compare the performance of various boron-containing aluminum-based materials to neutrons in radiotherapy, two radiotherapy scenarios were selected: proton radiotherapy and BNCT. To achieve the research objectives, this study was divided into several steps, as detailed below.

### 2.1. Secondary Neutron Spectra Simulations

**Figure 1:**
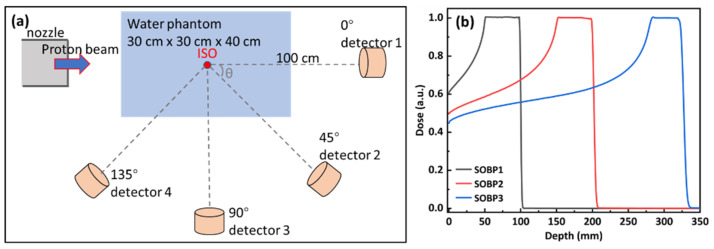
(**a**) Schematic diagram of the simulation setup for secondary neutrons produced by protons. (**b**) Three proton SOBPs with different modulation ranges were used as primary sources.

To obtain the secondary neutron spectra generated by radiotherapeutic protons, a detailed nozzle module of a proton and heavy ion accelerator, built in our previous work [19,20], was used. A water phantom (30 × 30 × 40 cm^3^) was positioned at the end of the beamline, and its geometrical center aligned with the isocenter of the accelerator. Four cylindrical detectors, each with a diameter of 229 mm and a length of 330 mm, consistent with the SWENDI-2 (Thermo Scientific, Waltham, MA, USA) neutron detector [21], were applied to record the secondary neutron spectral fluence. These detectors were placed at angles of 0°, 45°, 90°, and 135° relative to the beam axis, with their centers fixed at a distance of 100 cm from the isocenter. Figure 1a shows the schematic diagram of the simulation.

It should be mentioned here that the SWENDI-2 detector is cylindrical, with a diameter of 22.9 cm and a height of 21.2 cm. It utilizes polyethylene as a moderator and a ^3^He tube (2 atm) as the detector. The composites of the four simulated detectors of this study were set to air because the recorded neutron kinetics do not depend on the detector’s composition. Geant4 provides built-in methods for users to obtain the current position of particles and the volume they belong to. During the simulation, once a neutron is determined to have entered the volume of the detector, its kinetic energy is recorded. This differs somewhat from experimental measurements, where the measured neutron’s kinetic is inferred from the signal size produced by the neutron’s interaction with the detector material. This process is directly related to the detector material, as different materials have varying cross-sections for neutron collisions, resulting in different signal sizes.

Considering that the energy distribution of secondary neutrons depends on the energy of the primary protons, three proton spread-out Bragg peaks (SOBPs) with different modulation ranges were used as a primary source. These SOBPs were optimized and implemented in our previous work [20]. Each SOBP has a fixed modulation width of 50 or 40 mm, with centers located at depths of 75 (from 50 to 100 mm, denoted as SOBP1), 175 (from 150 to 220 mm, denoted as SOBP2), and 300 mm (from 280 to 320 mm, denoted as SOBP3), respectively, covering most clinical therapy needs. The integrated depth dose (IDD) curves for these SOBPs in water are shown in Figure 1b.

When a neutron entered a detector, its kinetic energy was recorded, and then it was immediately killed (stop tracking of the event in the simulation) because we were only interested in its kinetic energy upon entering the detector. A total of 1 × 10^8^ protons were simulated for each SOBP to ensure that statistical uncertainties in the dose calculations did not exceed 1%. After the simulations were completed, the neutrons recorded by each detector were tallied in 300 logarithmic energy bins, ranging from 10^−9^ MeV to 250 MeV to obtain the secondary spectrum at a specified detector angle.

### 2.2. Selection of Shielding Materials

Five types of boron-containing Al-based composites were selected as neutron shielding materials: Al-B_4_C, Al-BN, AlB_2_, Al-TiB_2,_ and Al-TiB_2_-BN. For convenience, each material is represented as Al-*x*M1-*y*M2, where *x* and *y* are the weight percent of additions M1 and M2, respectively. For the Al-*x*B_4_C, Al-*x*BN, Al-*x*B_2_, and Al-*x*TiB_2_ compositions, *x* values are 10, 20, 30, 40, and 50 wt.%. For the Al-*x*TiB_2_-*y*BN composition, *x* values are 10, 15, 20, and 30 wt.%, and corresponding *y* values are 40, 15, 30, and 20 wt.%, respectively. The weight percent of B in TiB_2_ was fixed at 32%, as it has been reported that the optimal mass fraction of Ti is about (2.2 × B + 1~1.5%). [12]. The simulated weight percentages of boron cover the ranges reported in the current literature. The shielding performance of concrete was also calculated, using it as a benchmark material for comparison and analysis. Its composition and density [22] are shown in Table 1.

### 2.3. Shielding Performance Assessment

The neutron shielding performance of different materials was evaluated in physical quantities, including ambient dose equivalent and dose recorded in the region of interest (ROI). Two types of neutron beams were used: one generated by protons and the other used in BNCT. These beams were set as primary sources and emitted vertically on the shielding material. The distance from the source to the upstream surface of the material was fixed at 100 cm, representing typical conditions for most facilities of proton and BNCT treatments. A spherical scoring volume 12 cm in diameter made of soft tissue-equivalent material (10.1 wt.% H, 11.1 wt.% C, 2.6 wt.% N, and 76.2 wt.% O, with a density of 1.0 g/cm^3^) was implemented to score the neutron spectral fluence after passing through the material. This sphere is recommended by the International Commission on Radiation Units and Measurements (ICRU) for use in radiation safety and dosimetry applications [23]. For each scenario (neutron source and specified material), various material thicknesses from 50 to 350 cm were simulated. The distance between the center of the ICRU sphere and the downstream surface of the shielding material was fixed at 30 cm. To ensure uniform neutron recording, the source was modeled as a circular plane with a diameter of 15 cm, slightly larger than the scoring volume. Neutrons were uniformly sampled from the source plane and incident perpendicular to the material. The center of the source and that of the ICRU sphere were aligned with each other along the neutron incident direction (overlapped from the beam eye’s view). Recorded quantities included neutron spectral fluence passing through the shielding material, neutron dose, γ dose, and total dose in the scoring volume. Neutron spectra were scored in units of cm^−2^ per incident proton. To obtain reliable statistical results for all scenarios, the number of simulated particles varied with material thickness: 1 × 10^7^ particles for thinner materials and 1 × 10^8^ for thicker materials.

Neutron spectral fluences recorded in the ICRU sphere were converted to the ambient dose equivalent, H*(10), using an in-house developed code and dose conversion factors provided by the International Committee of Radiological Protection (ICRP) publication 116 [24].

The ambient dose equivalent, H*(10), is calculated using the following equation [4,25]:

(1) $$H^{*}\left(10\right)=\sum_{i=1}^{N}\Phi_{i}\;*\;h_{i}$$

where *i* is the *i*th energy bin in the neutron spectral fluence, Φ*_i_* is the fluence in the *i*th energy bin, and *h_i_* is the corresponding conversion coefficient for that energy bin.

On the other hand, the total macroscopic cross-section, Σ*_t_*, of each shielding material was calculated using the well-known Beer–Lambert law [26]:

(2) $$I=I_{0}e^{-\sum_{t}x}$$

where *I* and *I*_0_ are the intensity of the neutrons recorded in the ICRU sphere when the shielding material was used or not, and *x* is the thickness of the shielding material in cm.

## 3. Results

### 3.1. Secondary Neutron Spectral Produced by Protons

**Figure 2:**
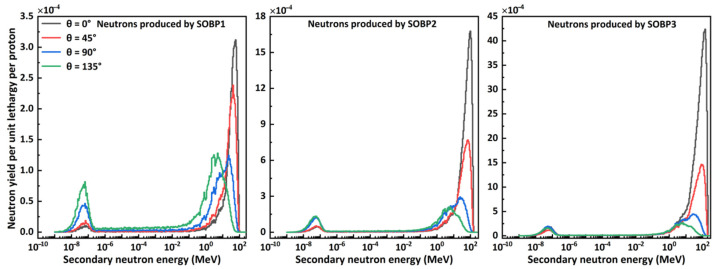
Secondary neutron spectral fluence produced by protons with different energies recorded at various detector angles. All figures share the same legend.

Figure 2 shows the secondary neutron spectral fluence produced by three proton SOBPs in water, as recorded by detectors placed at different locations. Each spectrum exhibits two distinct peaks: a low-energy peak, attributed to isotopic neutrons produced by evaporation processes, and a high-energy peak, associated with forward-peaked neutrons from intra-nuclear cascade reactions [4]. The ratio of the amplitude of the low-energy peak to that of the high-energy peak increases with the increasing angle between the detector and beam axis. The neutron spectra for a given SOBP are similar in shape, but as the angle increases, the amplitude of the low-energy peak increases, while that of the high-energy peak decreases, and the high-energy peak shifts towards the lower values. For different SOBPs, the energies of the high-energy peaks correlate directly with the proton energies that form the SOBP. For instance, at a 0° detector angle, the energies at the high-energy peaks are approximately 56.5 (SOBP1), 95.5 (SOBP2), and 135.5 MeV (SOBP3), respectively. Additionally, the amplitudes of both peaks increase with the primary proton energy.

Considering the energy and intensity distribution of the secondary neutron spectra, two typical spectra were selected for evaluating the shielding performance of materials. The first spectrum, produced by SOBP1 at 135° with an average energy of 14.90 MeV, and the secondary spectrum, with the maximum energy, produced by SOBP3 at 0° with an average energy of 109.24 MeV, were referred to as n-SOBP1 and n-SOBP3, respectively, as listed in Table 1.

**Figure 3:**
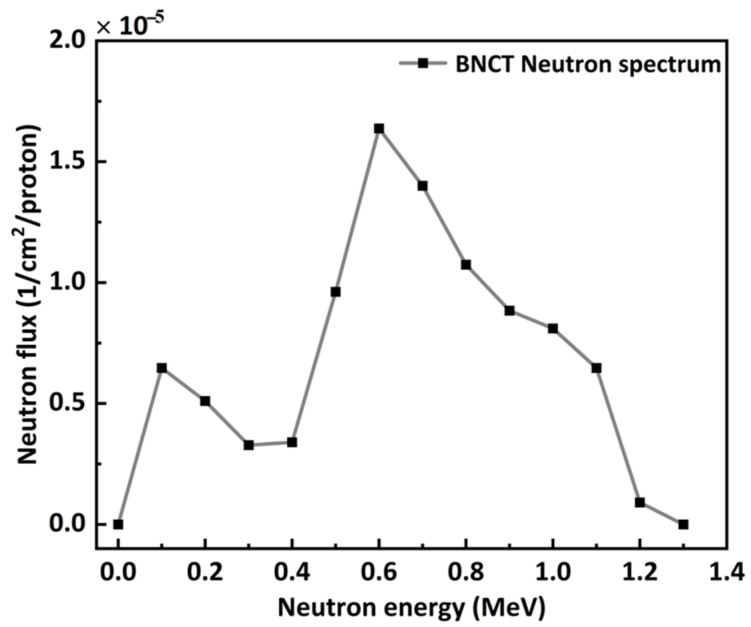
Neutron energy spectrum from the p-Li reaction at a proton energy of 2.8 MeV.

For BNCT scenarios, neutrons produced from different reactions serve as primary particles and have varying energies across different facilities. In this study, the neutron energy spectrum from the p-Li reaction at a proton energy of 2.8 MeV was selected as a primary source. This spectrum, shown in Figure 3, has an energy range from 0 to 1.3 MeV and is provided by Kiyanag [27].

As a result, three neutron spectra—two produced by protons and one used in BNCT—were utilized to study the shielding performance of materials. Their details are listed in Table 2.

### 3.2. Neutron Spectral Fluence After Passing Through Varying Materials

**Figure 4:**
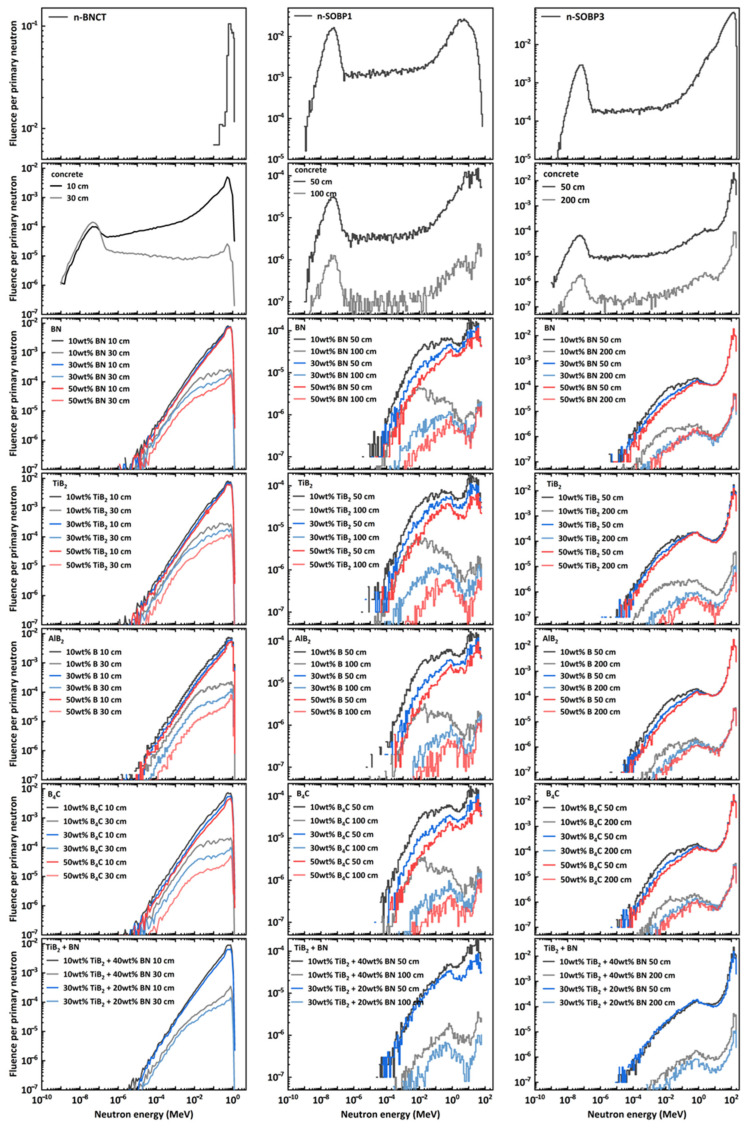
The neutron spectral fluence recorded in the ICRU sphere after passing through different shielding materials of various thicknesses. The first row shows the energy spectra of the three studied neutrons at the source plan.

Figure 4 shows neutron spectral fluence recorded in the ICRU sphere after passing through shielding materials of varying thicknesses. For convenience of comparison, the spectrals of the three studied scenarios at the source plane are displayed in the first row. The results are normalized to per primary neutron.

As demonstrated in the figure, boron-containing materials exhibit more efficient thermal neutron absorbers compared to concrete, especially for neutrons with energies below 10 keV, after passing through these boron-containing components, where almost none remain. On the other hand, the neutron fluence decreases, as expected, with increasing material thickness, either for concrete or for boron-content shielding materials. By comparing the neutron fluence distribution for a given material, it can be concluded that increasing the material’s thickness offers greater improvement in shielding performance than increasing the boron content. For instance, the data reveal that for neutrons with energies ranging from 10 keV to 100 MeV, when the material thickness is increased from 50 cm to 100 cm, the fluence at a specified energy decreases by approximately two orders of magnitude for neutrons produced by SOBP1. The same increased magnitude was also observed for neutrons produced by SOBP3 when the material thickness was increased from 50 cm to 200 cm. In contrast, for those neutrons, increasing the mass fraction of boron compounds from 10% to 50% only reduces the fluence at a certain energy by at most 2 to 3 times. Specially for some composites (e.g., Al-B4C, AlB2), the increase in boron content did not result in a reduction in the fluence of high-energy neutrons (around 100 MeV of n-SOBP3).

For the neutrons used in BNCT (as shown in the first column of Figure 4), the neutron fluence recorded after passing through boron-containing components is higher than that recorded after passing through concrete with the same thickness. This phenomenon contrasts with that observed for neutrons generated by protons. One possible reason is that the neutrons used in BNCT have lower energies (below 1.5 MeV, as shown in Figure 3). These energy range neutrons primarily losing energy through elastic collisions. Low atomic number elements, such as hydrogen, have a higher elastic cross-section. Concrete contains hydrogen, which is crucial for moderating these neutrons, resulting in better shielding effectiveness.

**Figure 5:**
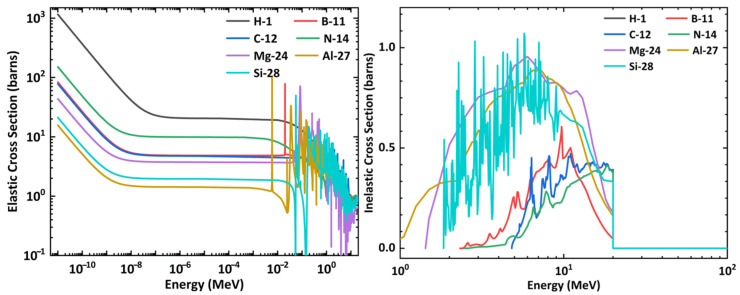
Elastic and inelastic collision cross sections of neutrons in different elements that constitute the shielding materials.

Figure 5 shows the elastic and inelastic collision cross sections of neutrons with various elements that constitute the shielding materials, based on data from the ENDF (Evaluated Nuclear Data File, https://www.nndc.bnl.gov/endf/index.html (accessed on 23 September 2024)). The elastic collision cross section for neutrons (with energy ranges from a few eV to 2 MeV) with hydrogen is significantly larger than that for other elements. This effectively explains why concrete, which contains hydrogen, provides better shielding performance for neutrons in this energy range compared to aluminum-based boron-containing materials.

Inelastic collisions occur only for neutrons with energies of at least 1 MeV, and the inelastic cross section of neutrons with Al is comparable to that of Si and Mg, which are the two main components of concrete.

Previous work [28] has also reported that concrete contains hydrogen, which allows it to effectively shield fast neutrons. Since neutrons and protons have almost equal masses, when a neutron collides with a hydrogen nucleus, the entire energy is transferred to the proton, resulting in the slowing down of the neutrons.

### 3.3. Neutron Ambient Dose Equivalent

For all boron-containing materials, the neutron spectral distribution and its variations with different boron content and increasing material thickness are nearly identical, as shown in Figure 4. Therefore, the neutron dose equivalent was used to quantify and compare their shielding performance.

**Figure 6:**
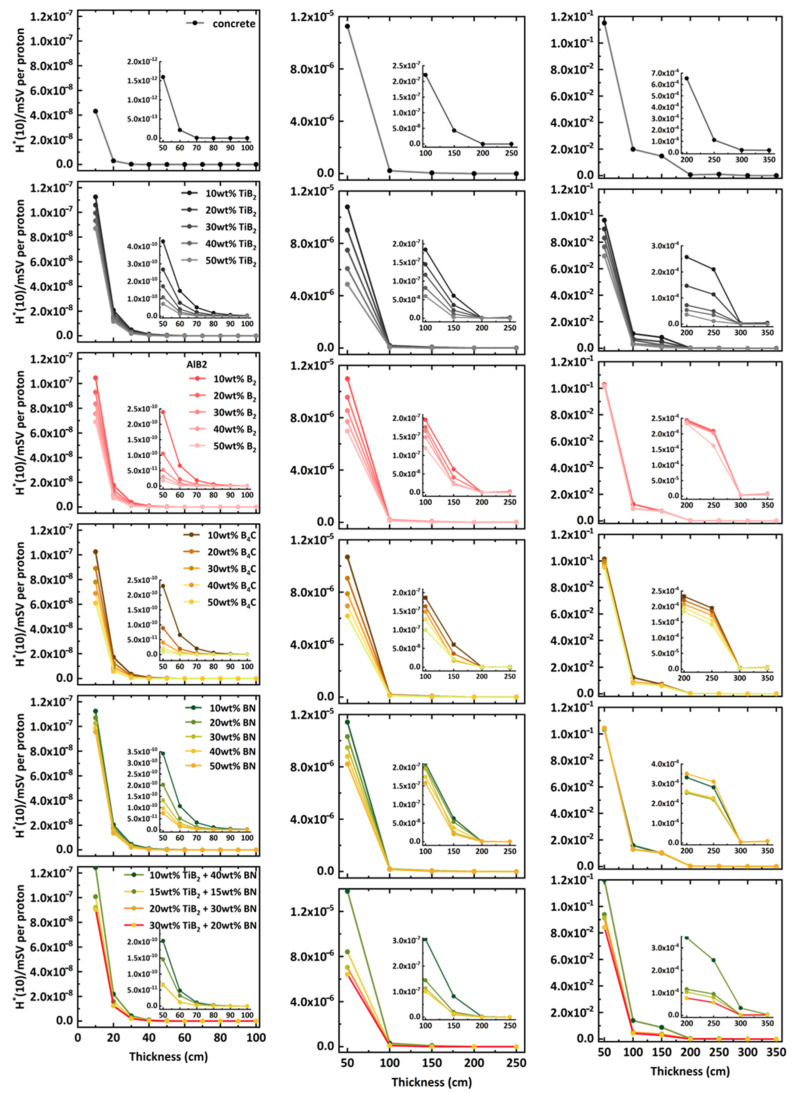
Neutron ambient dose equivalent values for different shielding materials. The left, middle, and right columns show the results for n-BNCT, n-SOBP1, and n-SOBP3, respectively. Each row uses the same legend.

Figure 6 shows the calculated neutron ambient dose equivalent values for different shielding materials, based on the neutron spectral fluence recorded in the ICRU sphere. All values were normalized to per incident proton. The left, middle, and right columns show the results for n-BNCT, n-SOBP1, and n-SOBP3, respectively.

For all shielding materials, the neutron ambient dose equivalent decreases sharply with increasing material thickness initially, then the reduction becomes more gradual with further increases in thickness. Once the thickness exceeds 70, 200, and 300 cm for the neutron spectra of n-BNCT, n-SOBP1, and n-SOBP3, respectively, the ambient dose equivalent value levels off, indicating that such thickness effectively shields against nearly all neutrons in these scenarios. For neutrons generated by protons (n-SOBP1 and n-SOBP3), all boron-containing materials demonstrate superior shielding performance compared to concrete with the same thickness. The neutron dose equivalent is reduced by at least approximately 50% at thicknesses of 100 and 150 cm compared to concrete for both n-SOBP1 and n-SOBP3. While for n-BNCT, the shielding performance of all boron-containing composites is inferior to that of concrete, which can be explained by the energy spectra shown in Figure 5. The neutrons have higher counts after passing through these composites than concrete because the hydrogen element in concrete moderates neutrons around the average energy of BNCT more efficiently. Moreover, for a specified material, increasing the boride compound content generally results in a lower dose equivalent value. By comparing materials with the same thickness and boron compound content, the following conclusions can be drawn: For n-SOBP1 and n-SOBP3, the neutron ambient dose equivalent decreases in the order Al-TiB_2_ > Al-B_4_C > AlB_2_ > Al-BN. For n-BNCT, the neutron dose equivalent decreases in the order Al-B_4_C > AlB_2_ > Al-TiB_2_ > Al/BN. For the Al-TiB_2_-BN composites with the same Al mass fraction, a higher TiB_2_ content results in a lower ambient dose equivalent; for example, the composite with 30 wt.% TiB_2_ and 20 wt.% BN has a lower ambient dose equivalent compared to the composite with 20 wt.% TiB_2_ and 30 wt.% BN.

### 3.4. Dose Distribution of Different Materials

**Figure 7:**
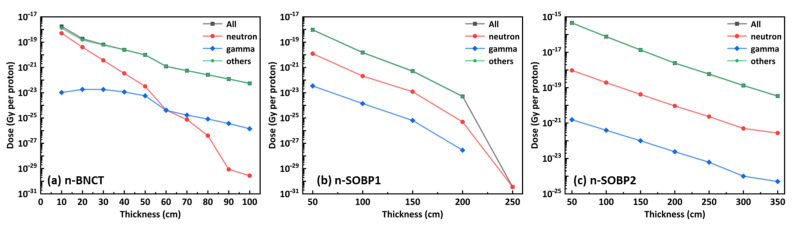
Dose distribution of different particles after passing through various thicknesses of concrete for the three studied neutron spectra.

Figure 7 shows the dose distribution of different particles recorded in the ICRU sphere after passing through varying thicknesses of concrete for the three studied neutron spectra. The doses were normalized per proton and categorized into three components: neutron dose, gamma dose, and others dose. The neutron and gamma doses represent the energy deposited as a result of collisions involving neutrons or photons directly participating in the interactions, respectively. While “others” dose refers to the dose contributed by secondary particles produced by neutrons (e.g., recoil nuclei from neutron inelastic collision) or by gamma rays (e.g., photoelectrons produced by the photoelectric effect), where neither neutrons nor gamma rays are directly involved in the energy deposition process. Since the dose distribution trends with increasing thickness for other materials are similar to those observed in concrete and, therefore, are not shown in the figure.

The dose distribution indicates that secondary particles contribute the most to the total dose, followed by neutrons, with gamma contributing the last. The dose contributions of neutrons and gammas are relative to the energies of the primary neutrons. For the n-BNCT, neutrons contribute approximately 25% to the total dose at a thickness of 10 cm, with their contribution gradually decreasing as thickness increases. For n-SOBP1 and n-SOBP3, neutrons contribute approximately 1% and 0.2% to the total dose, respectively, while gamma contributes approximately 3 × 10^−5^ and 4 × 10^−6^, respectively. Moreover, for the n-SOBP1, at a thickness of 250 cm, almost all the dose is contributed by neutrons, as gamma and secondary particles are almost absorbed by the material, leaving only neutrons with high initial kinetic energy to reach the scoring sphere.

It should be noted that we categorize energy deposition into three types based on the involvement of neutrons or gammas. Although the dose from secondary particles is the largest, this does not mean we can ignore neutrons. It is important to remember that these secondary particles, including gammas, are produced by neutrons through inelastic collisions with target material; they all originate from neutrons.

**Figure 8:**
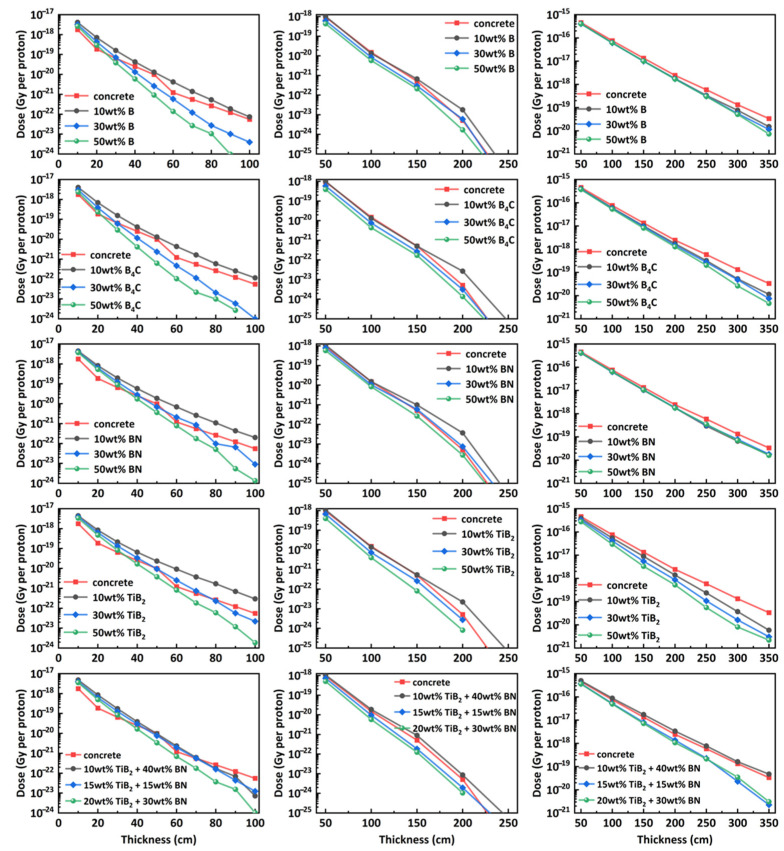
Comparison of total dose distribution for different materials at varying thicknesses and boron contents. The left, middle, and right columns present the results for n-BNCT, n-SOBP1, and n-SOBP3, respectively. Each row uses the same legend.

Figure 8 compares the total dose distribution with increasing thickness for various materials (first column: n-BNCT, middle column: n-SOBP1, right column: n-SOBP3) scored in the ICRU sphere. For convenience of comparison, the results of concrete are plotted in red in each subplot. It should be noted that for certain thicknesses, such as 90 cm for Al-50 wt.% B_4_C, total dose values are absent, as only a small number of neutrons with higher initial kinetic energy can pass through such thick material, and they are less likely to collide with the sphere to deposit energy.

The dose distribution clearly shows that, for composites of the same thickness as concrete, a lower total dose is achieved when the mass fraction of boron-containing compounds exceeds 10 wt.%, for neutrons generated by protons, and for n-BNCT when the material thickness exceeds 30 cm. The distribution also reveals that, for a given material at a specified thickness, increasing the mass fraction of a boron-containing compound from 10 wt.% to 30 wt.% results in a greater reduction in total dose compared to increasing it from 30% to 50% (especially for n-BNCT and n-SOBP1), even though both increments are by 20%. Moreover, as expected, the total dose increases with the average energy of the primary neutrons.

### 3.5. Neutron Macroscopic Cross-Sections

**Figure 9:**
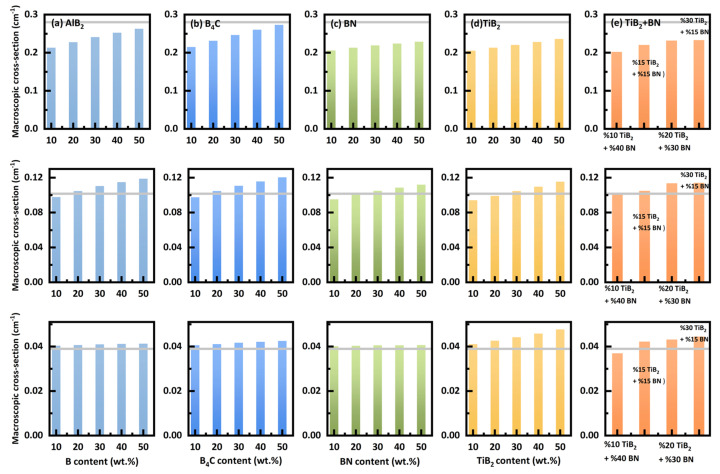
Neutron macroscopic cross-section for various materials with different boron contents at 10 cm for n-BNCT (**top row**) and 50 cm thickness for n-SOBP1 (**middle row**) and n-SOBP3 (**bottom row**). The gray line represents the results of concrete at the corresponding thickness. Each column uses the same legend.

Figure 9 shows the neutron macroscopic cross-section for various materials with different boron contents at a fixed thickness of 10 cm for n-BNCT (top row) and 50 cm for both n-SOBP1 (middle row) and n-SOBP3 (bottom row). For comparison, the cross-section value of concrete at the same thickness is plotted as a gray line. As expected, the cross-sections increase with boron content for all three studied neutron spectra. For n-BNCT, the macroscopic cross-sections of all materials with any boron content are lower than that of concrete. In contrast, for n-SOBP1, when the boron-containing compound exceeds 30 wt.%, the macroscopic cross-sections of all materials become larger than that of concrete at the same thickness. For n-SOBP3, however, a composite with 10 wt.% boron compound content has a larger cross-section than the same concrete at the same thickness. This trend is consistent with the changes observed in the ambient dose equivalent and dose.

## 4. Discussion

In this study, we evaluated the shielding performance of several boron-containing Al-based materials for neutrons in proton therapy and BNCT settings. In these treatment facilities, concrete is commonly used as shielding material. However, due to the high energy and longer range of neutrons in these environments, several meters of concrete are required to meet shielding requirements. Therefore, improving shielding efficiency and reducing the thickness of the shielding material is crucial, particularly as many additional proton and heavy ion therapy facilities are being planned and constructed. Recently, boron-containing Al-based materials (e.g., Al-B_4_C, AlB_2_) have been widely studied as potential replacements for concrete in neutron shielding applications. However, boron content significantly affects the microstructure of the base material. For B/Al alloy, a previous study [11] has reported that adding boron to Al tends to form boron-rich boride at the grain boundaries, which reduces the toughness of the alloy. It is well known that a high B/Al alloy containing 10 wt.% to 50 wt.% boron has an excellent neutron absorption capability; however, higher boron content also results in poor mechanical properties. For the Al-B_4_C composite, the B_4_C content can reach up to 50 wt.%, and it generally ranges from 5 wt.% to 50 wt.%. However, the microhardness of the composite decreases when the B_4_C ratio exceeds a certain threshold [7]. Therefore, in this study, the mass fraction of boron-containing compounds was set between 10 wt.% and 50 wt.%.

Among the boron-containing Al-based materials studied in this study, Al/B_4_C is the most common one, which has been widely reported in previous works and manufactured using fabrication methods [15,16]. Unlike these works, which primarily focus on evaluating the shielding performance of Al/B4C against thermal neutrons [15,16], this study investigates its shielding performance against neutrons with a broader energy distribution range. Moreover, this study employs more physical quantities, such as neutron ambient dose equivalent, cross-section, and energy spectrum, to systematically evaluate the shielding performance of these materials. By mainly applying MC simulations (theoretical calculations), this study allows for the assessment and comparison of different types of boron-containing materials simultaneously. Due to constraints such as experimental conditions, manufactures or measurements are often limited to a single material. However, manufacturing processes and mechanical properties are also crucial parameters for shielding materials, directly affecting their practical application. Therefore, in subsequent work, we will conduct experiments to study these parameters.

In our MC simulations, the shielding materials are assumed to be homogeneous, which may not fully reflect practical situations. In reality, factors such as manufacturing processes and material properties often result in shielding materials that are not perfectly homogeneous, either in density or in the distribution of added particles. However, determining the inhomogeneity of materials theoretically is challenging. Even for two samples of the same material produced under identical conditions, their uniformity can vary. Therefore, it is necessary to assume an ideal homogeneous distribution of materials when calculating their shielding performance.

In proton therapy, several types of secondary particles are generated through the nonelastic collision of protons with matter, including charged ions (e.g., helium, deuterium, tritium) and uncharged particles (e.g., gamma, neutron). These secondary ions are relatively easy to shield, requiring only a few tens of centimeters of concrete to completely absorb. As shown in Figure 1, a 230 MeV proton has a range of approximately 32 cm in water. Therefore, the primary focus of shielding in proton therapy is on uncharged particles, particularly neutrons. This study only evaluates the shielding performance of materials for neutrons, but future work should consider gamma shielding, as gamma is a non-neglect byproduct in proton therapy [29,30].

The secondary neutron spectral fluence shown in Figure 3 indicates that the shape of the spectrum depends not only on the angle relative to the beam axis but also on the energy of the primary incident proton. High-energy neutron fluences, which are the primary concern for shielding, are greatest along the beam’s central axis. This suggests that if a material meets the shielding requirements at the beamline end, it will also meet the requirements in other directions. Moreover, the simulated neutron spectral fluence aligns with previous studies [4], which have demonstrated that the neutron ambient dose equivalent does not significantly change with angle relative to the beam axis in the downstream region. Therefore, when studying neutron shielding in proton therapy, it is sufficient to focus on the shielding performance along the beam’s central axis.

Compared to concrete, boron-containing materials exhibit stronger absorption capabilities for thermal neutrons, as shown in Figure 4. When the boron content exceeds a certain threshold, these materials offer better shielding performance, demonstrated by lower neutron ambient dose equivalent (Figure 6) and total dose (Figure 8), as well as a higher macroscopic cross-section (Figure 9). The improved shielding performance might be attributed to the wider energy range of secondary neutrons produced by protons, from thermal to a hundred MeV. The proportion of thermal neutrons is not negligible, especially at larger angles relative to the beam axis for lower-energy incident protons. On the other hand, as neutrons pass through the shielding material, their energy decreases through collisions, continuously generating low-energy neutrons. The presence of boron in the material efficiently absorbs these thermal neutrons, resulting in dose reduction and increased cross-section.

For neutrons generated in proton therapy, this study only considers those generated in a phantom, which differs from typical patient treatment scenarios. In actual proton radiotherapy, neutrons can be generated from three processes [31,32,33]: (1) Production in the cyclotron, such as from proton collisions with the cyclotron walls or magnet; (2) product in the beam delivery nozzle, involving components like the range modulator wheel, ionization chambers, and window; and (3) production in the patient. Therefore, when fully simulating the proton transport process in radiotherapy, more neutrons will be produced, and more efficient shielding materials might be needed. Future work should consider neutrons produced from these various sources and processes.

## 5. Conclusions

This study evaluated the shielding performance of several boron-containing Al-based materials for neutrons in two scenarios: proton radiotherapy and BNCT. A total of five materials were selected, including AlB_2_, Al-B_4_C, Al-TiB_2_, Al-BN, and Al-TiB_2_-BN, each with boron compound mass fraction ranging from 10 wt.% to 50 wt.%. Neutrons generated in these two settings were set as the source. To assess and compare the shielding performance of those materials, three physical qualities—neutron ambient dose equivalent, dose contribution from different particles, and neutron macroscopic cross-section—were calculated. The results indicated that, compared to concrete of the same thickness, the absorption capacity of boron-containing materials for thermal neutrons is significantly enhanced. For neutrons generated by protons, when the mass fraction of boron compounds exceeds 30 wt.%, these materials exhibit better shielding performance than concrete, as evidenced by lower neutron ambient dose equivalents, lower dose values, as well as higher macroscopic cross-section. However, for neutrons used in BNCT, the shielding performance of all composites is inferior to that of concrete, as the hydrogen element in concrete moderates neutrons around the average energy of BNCT more efficiently. For a given material, its shielding capability increases with the boron compound content. Among the five materials when the thickness and boron compound content are the same, the shielding performance for neutrons generated by protons, from best to worst, is as follows: Al-TiB_2_, Al-B_4_C, AlB_2_, Al-TiB_2_-BN, and Al-BN. While for BNCT, the shielding performance from best to worst is in the order: Al-B_4_C, AlB_2_, Al-TiB_2_, Al-TiB_2_-BN, and Al-BN. In the next step, a more systematic computational evaluation of these shielding materials is needed to assess their performance more comprehensively. Additionally, some measurements should be conducted to validate the MC estimates.

## Figures and Tables

**Table 1:** Concrete composition.

Density	2.35 g cm^−3^
Mass fraction (%)	H (1.0), C (0.1), Mg (52.91), Al (3.9), Si (33.7), Ca (4.4), K (1.3), Na (1.6), Fe (1.4)

**Table 2:** Neutron spectra utilized in this work to study the shielding performance of the materials.

Label	Energy (MeV)			Description
	Min	Max	Average	
n-BNCT	0	1.30	0.65	Neutron spectrum from the p-Li reaction and unitized in BNCT
n-SOBP1	1.0 × 10^−9^	67.30	14.90	Neutrons generated by SOBP1, and scored at 135°
n-SOBP3	1.0 × 10^−9^	209.87	109.24	Neutrons generated by SOBP3, and scored at 0°

## Data Availability

The data that support the findings of this study are available from the corresponding author upon reasonable request.
